# Supplementary figures and images for: Distinct mechanisms of type 3 secretion system recognition control LTB4 synthesis in neutrophils and macrophages
Source: PLoS Pathog. 2024 Oct 18;20(10):e1012651. doi: 10.1371/journal.ppat.1012651 (PMC11524448; doi:10.1371/journal.ppat.1012651)

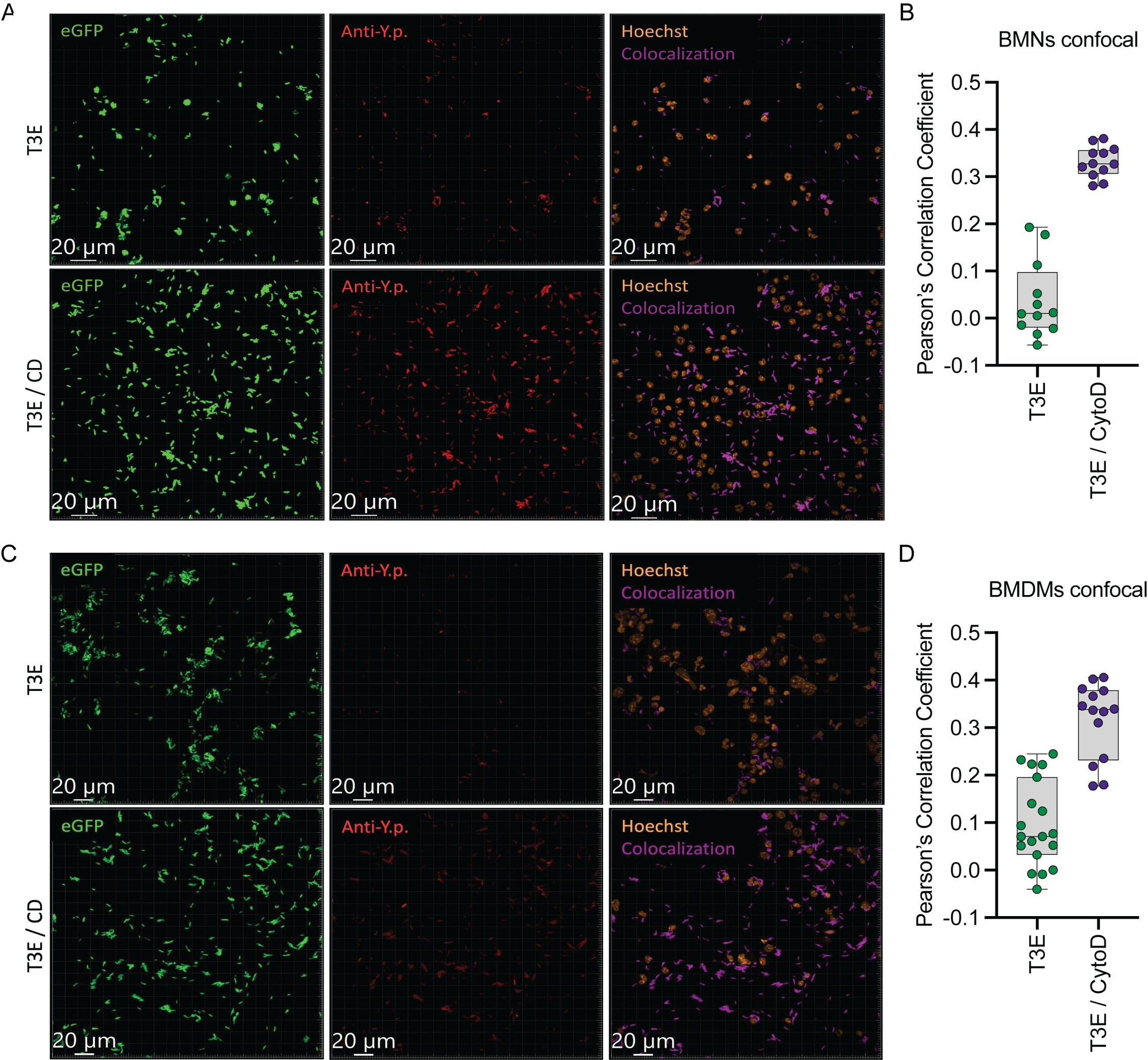

Supplement: S1 Fig — (A-B) BMNs or (C-D) BMDMs were either left untreated (T3E) or pre-treated with cytochalasin D (10 μM; T3E / CD) for 30 min prior to infection with Y. pestis T3E at an MOI of 10 for (A-B) 1 h or (C-D) 4 h. (A,C) Representative confocal images of 3 biological replicates; eGFP = intracellular bacteria; Anti-Y.p. = extracellular bacteria. (B,D) Pearson scores calculated for eGFP and Anti-Y.p.-Alexa647 from 3 biological replicates, 3–4 images collected from each replicate. (TIF) [file ppat.1012651.s001.tif]

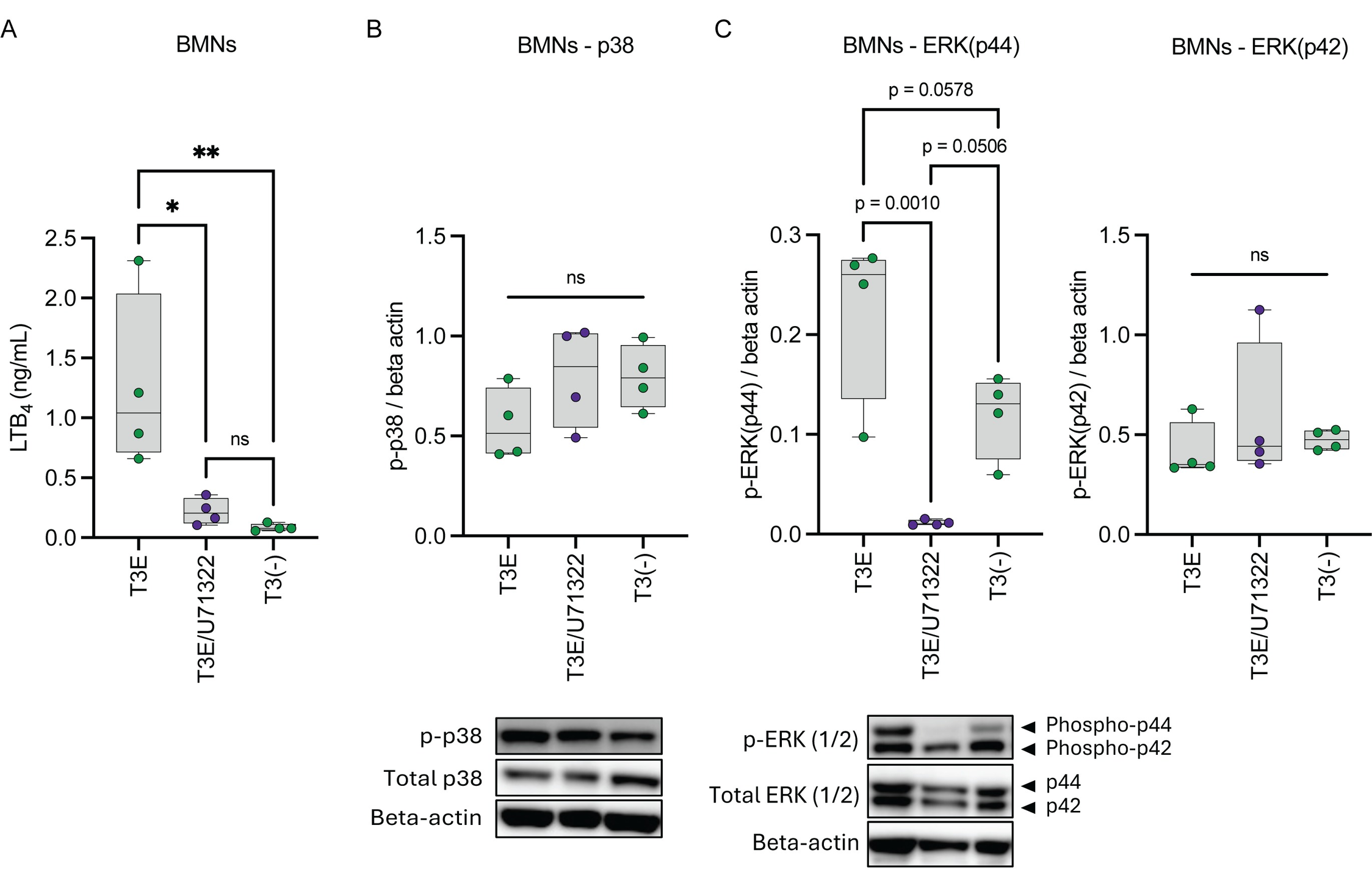

Supplement: S2 Fig — BMNs were either UT (green circles) or treated with PLC inhibitor (purple circles) prior to infection with Y. pestis T3E or Y. pestis T3(-) at an MOI of 20 for 1 h. (A) LTB4 was measured from supernatants by ELISA. Densitometry and representative WB images for (B) p-p38 or (C) p-ERK (p44 and p42) from whole cell lysates normalized to beta actin. (A-C) Each symbol represents an independent biological infection, and the box plot represents the median of the group ± the range. UI = uninfected. ns = not significant. One-way ANOVA with Tukey’s post hoc test compared to each condition. * = p≤0.05, ** = p≤0.01, **** = p≤0.0001. (TIF) [file ppat.1012651.s002.tif]
