# Supplementary material for: Distinct mechanisms of type 3 secretion system recognition control LTB4 synthesis in neutrophils and macrophages
Source: PLoS Pathog. 2024 Oct 18;20(10):e1012651. doi: 10.1371/journal.ppat.1012651 (PMC11524448; doi:10.1371/journal.ppat.1012651)
Supplement: S1 Table — (DOCX) [file ppat.1012651.s003.docx]

| **Name in manuscript** | **Genotype** | **Strain ref. #** | **Source** |
| --- | --- | --- | --- |
| **Bacteria** |  |  |  |
| *Y. pestis* | KIM1001 pgm-, pMT1+, pPCP1+, pCD1+, pML001+ | JG598 | [96] |
| *Y. pestis* T3^(-)^ | KIM1001 pgm-, pMT1+, pPCP1+, pCD1-, pML001+ | JG597 | [96] |
| *Y. pestis* T3E, eGFP | KIM1001 pgm-, pMT1+, pPCP1+, pCD1+ (yopH^Δ3-467^ yopE^Δ40-197^ yopK^Δ4-181^ yopM^Δ3-408^ ypkA^Δ3-731^ yopJ^Δ4-288^ yopT^Δ3-320^), pGEN222+ | YPA366 | This work |
| *Y. pestis* T3E | KIM1001 pgm-, pMT1+, pPCP1+, pCD1+, (yopH^Δ3-467^ yopE^Δ40-197^ yopK^Δ4-181^ yopM^Δ3-408^ ypkA^Δ3-731^ yopJ^Δ4-288^ yopT^Δ3-320^), pML001+ | JG715 | [96] |
| *Y. pestis* T3E +ypkA | KIM1001 pgm-, pMT1+, pPCP1+, pCD1+ (yopH^Δ3-467^ yopE^Δ40-197^ yopK^Δ4-181^ yopM^Δ3-408^ yopJ^Δ4-288^ yopT^Δ3-320^), pML001+ | JG684 | [96] |
| *Y. pestis* T3E +yopE | KIM1001 pgm-, pMT1+, pPCP1+, pCD1+ (yopH^Δ3-467^ yopK^Δ4-181^ yopM^Δ3-408^ ypkA^Δ3-731^ yopJ^Δ4-288^ yopT^Δ3-320^), pML001+ | JG681 | [96] |
| *Y. pestis* T3E +yopH | KIM1001 pgm-, pMT1+, pPCP1+, pCD1+ (yopE^Δ40-197^ yopK^Δ4-181^ yopM^Δ3-408^ ypkA^Δ3-731^ yopJ^Δ4-288^ yopT^Δ3-320^), pML001+ | JG680 | [96] |
| *Y. pestis* T3E +yopJ | KIM1001 pgm-, pMT1+, pPCP1+, pCD1+ (yopH^Δ3-467^ yopE^Δ40-197^ yopK^Δ4-181^ yopM^Δ3-408^ ypkA^Δ3-731^ yopT^Δ3-320^), pML001+ | JG686 | [38] |
| *Y. pestis* T3E +yopK | KIM1001 pgm-, pMT1+, pPCP1+, pCD1+ (yopH^Δ3-467^ yopE^Δ40-197^ yopM^Δ3-408^ ypkA^Δ3-731^ yopJ^Δ4-288^ yopT^Δ3-320^), pML001+ | JG682 | [96] |
| *Y. pestis* T3E +yopM | KIM1001 pgm-, pMT1+, pPCP1+, pCD1+ (yopH^Δ3-467^ yopE^Δ40-197^ yopK^Δ4-181^ ypkA^Δ3-731^ yopJ^Δ4-288^ yopT^Δ3-320^), pML001+ | JG683 | [96] |
| *Y. pestis* T3E +yopT | KIM1001 pgm-, pMT1+, pPCP1+, pCD1+ (yopH^Δ3-467^ yopE^Δ40-197^ yopK^Δ4-181^ yopM^Δ3-408^ ypkA^Δ3-731^ yopJ^Δ4-288^), pML001+ | JG685 | [96] |
| *Y. pestis* T3E *yopB* | KIM1001 pgm-, pMT1+, pPCP1+, pCD1+ (yopH^Δ3-467^ yopE^Δ40-197^ yopK^Δ4-181^ yopM^Δ3-408^ ypkA^Δ3-731^ yopJ^Δ4-288^ yopT^Δ3-320^yopB ^Δ7-396^), pML001+ | YPA322 | [38] |
| *Y. pestis* *yopB*::c*yopB* | KIM1001 pgm-, pMT1+, pPCP1+, pCD1+ (yopH^Δ3-467^ yopE^Δ40-197^ yopK^Δ4-181^ yopM^Δ3-408^ ypkA^Δ3-731^ yopJ^Δ4-288^ yopT^Δ3-320^), pML001+ | YPA362 | [38] |
| *S.* Typhimurium | *Salmonella* *enterica* Typhimurium LT2 pGENLux - 14028s | LOU120 | [38] |
| *S.* Typhimurium  SPI-1 null mutant | *Salmonella* *enterica* Typhimurium *invA::Km* - 14028s | MJW1301 | [82] |
| *S.* Typhimurium  SPI-2 null mutant | *Salmonella* *enterica* Typhimurium *ssak::Cm* - 14028s | MJW1835 | [97] |
| *S.* Typhimurium  SPI-1/2 mutant | *Salmonella* *enterica* Typhimurium *invA::Km ssak::Cm* - 14028s | MJW1836 | Micah Worley |
| **Plasmids** |  |  |  |
| pGEN222 | GFP gene | NA |  |
| pML001 | Luciferase bioreporter | NA | [96] |
